# An *Msx2*-*Sp6-Follistatin* Pathway Operates During Late Stages of Tooth Development to Control Amelogenesis

**DOI:** 10.3389/fphys.2020.582610

**Published:** 2020-10-26

**Authors:** Intan Ruspita, Pragnya Das, Yan Xia, Sarah Kelangi, Keiko Miyoshi, Takafumi Noma, Malcolm L. Snead, Rena N. D’Souza, Marianna Bei

**Affiliations:** ^1^Center for Regenerative and Developmental Biology, The Forsyth Institute, Cambridge, MA, United States; ^2^Department of Prosthodontics, Universitas Gadjah Mada, Yogyakarta, Indonesia; ^3^Division of Neonatology, Cooper University Hospital, Camden, NJ, United States; ^4^Center for Engineering in Medicine, Department of Surgery, Massachusetts General Hospital and Harvard Medical School, Boston, MA, United States; ^5^Shriners Hospital for Children, Boston, MA, United States; ^6^Department of Molecular Biology, Institute of Biomedical Sciences, Tokushima University, Tokushima, Japan; ^7^Faculty of Human Life Studies, Hiroshima Jogakuin University, Hiroshima, Japan; ^8^Center for Craniofacial Molecular Biology, Herman Ostrow School of Dentistry of USC, University of Southern California, Los Angeles, CA, United States; ^9^University of Utah, Salt Lake City, UT, United States

**Keywords:** *Msx2*, *Sp6*, *follistatin*, dental epithelial cells, *in situ* hybridization, chromatin immunoprecipitation

## Abstract

**Background:**

Ameloblasts are epithelially derived cells responsible for enamel formation through a process known as amelogenesis. Amongst the several transcription factors that are expressed during amelogenesis, both *Msx2* and *Sp6* transcription factors play important role. *Msx2* and *Sp6* mouse mutants, exhibit similar amelogenesis defects, namely enamel hypoplasia, while humans with amelogenesis imperfecta (AI) carry mutations in the human homologues of MSX2 or SP6 genes. These across species similarities in function indicate that these two transcription factors may reside in the same developmental pathway. In this paper, we test whether they work in a coordinated manner to exert their effect during amelogenesis.

**Methods:**

Two different dental epithelial cell lines, the mouse LS8 and the rat G5 were used for either overexpression or silencing of *Msx2* or *Sp6* or both. *Msx2* mutant mouse embryos or pups were used for *in vivo* studies. *In situ* hybridization, semi-quantitative and quantitative real time PCR were employed to study gene expression pattern. *MatInspector* was used to identify several potential putative *Msx2* binding sites upstream of the murine *Sp6* promoter region. Chromatin Immunoprecipitation (chIP) was used to confirm the binding of *Msx2* to *Sp6* promoter at the putative sites.

**Results:**

Using the above methods we identified that (i) *Msx2* and *Sp6* exhibit overlapping expression in secretory ameloblasts, (ii) *Sp6* expression is reduced in the *Msx2* mouse mutant secretoty ameloblasts, and (iii) that *Msx2*, like *Sp6* inhibits *follistatin* expression. Specifically, our *loss-of* function studies by silencing *Msx2* and/or *Sp6* in mouse dental epithelial (LS8) cells showed significant downregulation of *Sp6* but upregulation of *Fst* expression. Transient transfection of *Msx2* overexpression plasmid, up-regulated *Sp6* and downregulated *Fst* expression. Additionally, using *MatInspector*, we identified several potential putative *Msx2* binding sites, 3.5 kb upstream of the murine *Sp6* promoter region. By chIP, we confirmed the binding of *Msx2* to *Sp6* promoter at these sites, thus suggesting that *Sp6* is a direct target of *Msx2*.

**Conclusion:**

Collectively, these results show that *Sp6* and *Msx2* work in a concerted manner to form part of a network of transcription factors that operate during later stages of tooth development controlling ameloblast life cycle and amelogenesis.

## Introduction

The development of teeth depends on cell interactions between epithelium and mesenchyme that leads to the differentiation of cells derived from mesenchyme into odontoblasts and of cells derived from epithelium into ameloblasts (Kollar and Lumsden, 1979; Thesleff and Nieminen, 2005). The process of epithelial cells differentiating into functional ameloblasts is time-dependent and through this process several morphologic changes occur known as: (i) *the inductive stage* (pre-ameloblasts); (ii) *the initial-secretory stage*; (iii) *the secretory stage*; and (iv) the *maturation stage* (Nanci, 2007). Pre-secretory, secretory, and mature ameloblasts express several proteins, including secreted proteins, enzymes, signaling molecules, cell–cell adhesion molecules, and transcription factors (reviewed in Wright et al., 2000, 2015; Aldred et al., 2003; Wright, 2006; Hu et al., 2008; Nakamura et al., 2008; Bei, 2009a,b; Bartlett, 2013; Habelitz, 2015; Shin et al., 2020).

Studies in animal models and humans have shown that Msx2 and Sp6 transcription factors play important role during amelogenesis. Specifically, in mice lacking the homeobox gene *Msx2* “the ameloblasts reach the secretory stage of their differentiation process, but only sparse amounts of enamel matrix are deposited” (Satokata et al., 2000; Bei et al., 2004; Babajko et al., 2014). In a case of syndromic amelogenesis imperfecta (AI) sequence analysis of the human homolog of MSX2 gene identified a missense mutation of T447C, further indicating the important role of *Msx2* during amelogenesis (Suda et al., 2006). Expression studies have also indicated that Msx2 is required for the expression of important secreted proteins and cell-cell adhesion molecules for amelogenesis, such as *laminin 5 alpha 3*, *amelogenin*, and *enamelin* (Bei et al., 2004; Ruspita et al., 2008; Molla et al., 2010).

Specificity protein 6 (*Sp6*) is another transcription factor that, like *Msx2*, is expressed by secretory ameloblasts and when its function is eliminated in mice amelogenesis is affected. The *Sp6* mutant mice among other phenotypes exhibit enamel hypoplasia (Nakamura et al., 2008; Utami et al., 2011). Recently, in a Caucasian family with autosomal dominant hypoplastic AI, a missense protein change, p.(Ala273Lys), is identified in *SP6*, the gene encoding the SP6 transcription factor (Smith et al., 2020). The authors have also “identified a potential SP6 binding motif in the *AMBN* proximal promoter sequence and showed that wild-type (WT) SP6 binds more strongly to it than the mutant protein,” further indicating the important role of Sp6 in amelogenesis (Smith et al., 2020). Earlier studies indicate that *Sp6* promotes amelogenesis *in vitro* through inhibition of *follistatin* (*Fst*) gene which is a soluble extracellular inhibitor of TGFβ superfamily and is involved in differentiation of secretory ameloblasts during tooth development (Ruspita et al., 2008). In addition, “overexpression of *follistatin* in the dental epithelium inhibits ameloblast differentiation in transgenic mouse incisors, whereas in *follistatin* knockout mice, ameloblasts differentiate ectopically on the lingual enamel-free surface” (Wang et al., 2004).

Based on the above, the role of *Msx2* and *Sp6* genes in amelogenesis is important. Here, we study the interplay between *Msx2, Sp6*, and *Fst* and we show that (i) *Sp6* expression is reduced in the *Msx2* mouse mutant secretoty ameloblasts, (ii) *Msx2*, like *Sp6* inhibits *follistatin* expression *in vitro*, (iii) *Sp6* and *follistatin* are early response genes whose expression is under the control of Msx2, and that (iv) Msx2 binds to *Sp6* promoter *in vitro*, suggesting that *Sp6* is a direct target of *Msx2*. These results raise the possibility that these transcription factors interact closely with each other and within a common molecular cascade.

## Materials and Methods

### Cell Culture

Two different dental epithelial cell lines were used in the present study – the rat dental epithelial cell line (G5), generously provided by Dr. Takafumi Noma, Dental School of Tokushima University, Japan and the mouse dental epithelial cell line (LS8) kindly provided by Dr. Malcolm Snead, USC, CA, United States. Both cell lines were maintained in high-glucose Dulbecco’s modified Eagle’s medium (Gibco, Grand Island, NY, United States), containing 100 U/ml penicillin, 100 mg/ml streptomycin, and 10% fetal bovine serum (Gibco, Grand Island, NY, United States) at 37°C in 5% CO_2_ humidified atmosphere following the standard protocols (Ruspita et al., 2008; Chang et al., 2017)

### Gain-of-Function and Loss-of-Function Studies

For overexpression of *Msx2*, LS8, and G5 cells were transfected with pCMVtag2-Flag-*Msx2*, and then cultured for 48–72 h following which total RNA was isolated from the cells using Trizol (Qiagen, MD, United States). An empty vector (pCMVtag2) served as a negative control for gain-of-function studies.

For loss of function of *Msx2*, commercially available small interfering RNA for *Msx2* (*Msx2*-siRNA) was purchased from Santa Cruz Biotechnology (Santa Cruz, CA, United States). We used the following oligonucliotides, sense sequence 5′CAGCUCUCUGAACCUUAC 3′ (sc-43947). As negative control we used a scramble sequence that will not lead to the specific degradation of any known cellular mRNA: sense scramble control 5′UUCUCCGAACGUGUCACG 3′ (sc-37007). To prepare lipid-siRNA complexes, the siRNA (80 pmol) in 100 μl of transfection medium (sc-36868) and 5 μl of siRNA transfection reagent (sc-29528) in 100 μl of transfection medium were combined, incubated for 30 min at 25°C, and then diluted with 800 μl of transfection medium. Cells were rinsed once with serum-free DMEM/F12, and 1000 μl of lipid-siRNA mixture-described above-was applied per well. After incubation for 6 h at 37°C in a humidified 5% CO_2_ cell chamber, an additional 1 ml of 20% FBS in DMEM/F12 was added per well, and lipofection was allowed to continue overnight. The following day, lipofection media was aspirated, and transfected monolayer cells refed with fresh 10% FBS in DMEM/F12. After 48 and 72 h, total cellular RNA was harvested for reverse-transcriptase-polymerase chain reaction (RT-PCR) analysis. For lentiviral (ShRNA) gene knock down assay, we obtained plasmids containing the sequences for *Msx2*-shRNA, *Sp6-shRNA* and non-target scramble shRNA from Sigma Aldrich (St. Louis, MO, United States). LS8 cells were seeded into six-well culture plates and cultured in DMEM/F12 containing 10% fetal bovine serum without antibiotic. Upon 80% confluency, cells were infected with lentiviruses with a MOI (multiplicity of infection) = 5 and selected for stable integration with 1 μg/ml puromycin.

### Time Dependent Assay

LS8 cells were transfected with pCMVtag2-Flag-*Msx2* (Invitrogen, United States) and then cultured for up to 48 h. The cells were harvested at 4 and 16 h for RNA isolation and subjected to real time qPCR analysis to check for expression of *Sp6* and *Fst*.

### RNA Extraction and Reverse-Transcriptase-Polymerase Chain Reaction

Total RNA was isolated from cultured cells after the desired time points using the standard procedure by TRIZOL (Qiagen) method. First-strand cDNA was generated from 1 μg of total RNA using quantitect, RT kit (Qiagen, Valencia, CA, United States) in total of 20 μl according to the manufacturer’s instructions. Semi-quantitative PCR was performed on 1 μl of RT product in 20 μl of reaction mixture to check for expression of *Msx2, Sp6*, and *Fst. Gapdh* was used as the loading control. The PCR products were analyzed on 1.5% agarose gel. The primer sequences are listed in Supplementary Table 1.

### Real-Time Quantitative PCR

For real-time quantitative PCR, total RNA from was isolated from cultured cells after the desired time points using the standard procedure by TRIZOL (Qiagen) method and reverse transcriptions were performed using qScript cDNA synthesis kit (Quanta Biosciences, Gaithersburg, MD, United States). Quantitative PCR was carried out in LightCycler and LightCycler-Faststart DNA Master SYBR Green I (Roche Diagnostics, Switzerland). The expression level of each sample was normalized to glyceraldehyde-3phosphate dehydrogenase (GAPDH) mRNA expression. The primer sequences are listed in Supplementary Table 1.

### Mice and Genotyping

All animal studies and experimental procedures were conducted in accordance to the guidelines for the care and use of laboratory animals by the Forsyth Institute, Cambridge, MA and Massahusetts General Hospital, Boston, MA. Embryos and postnatal pups (E18.5, P1 and P3) were collected from matings of *Msx2* heterozygous animals maintained in BALB/c background. The day of plug discovery was designated as embryonic day 0.5 (E0.5). Genotyping was performed as previously described (Bei et al., 2004). Age matched wildtype pups and/or embryos served as the appropriate controls.

### *In situ* Hybridization

Embryonic Day 18.5 embryos and postnatal animals (P1, P3) were collected and heads decapitated for making coronal and sagittal sections. E18.5, P1 and P3 samples were immediately fixed in 4% paraformadehyde. All samples were then dehydrated through graded ethanol series, embedded in paraffin, sectioned at 8 μm and processed for in situ hybridization (ISH), as previously described (Bei and Maas, 1998). Murine *Sp6* and *Fst* antisense probes were purchased from IDT (IA, United States) and labeled with DIG-UTP (Roche) following the manufacturer’s instructions. The sense probes for both genes were used as a negative control. *In situ* hybridization was performed as previously described (Bei et al., 2004)

### Chromatin Immunoprecipitation Assay

Chromatin immunoprecipitation (chIP) was performed using the EZ-Magna chip kit (Millipore, Billerica, MA, United States) according to the manufacturer’s instructions. Forty-eight hours after transfection with pCMV-FLAG-*Msx2* expression plasmid, LS8 cells were fixed and crosslinked with 1% (v/v) formaldehyde at 37°C for 10 min. Crosslinking was stopped by adding glycine to a final concentration of 125 mM, followed by washing with cold PBS. After sonication chromatin was incubated with magnetic beads conjugated to either 1 μg of monoclonal anti-Flag antibody (Sigma) or 1 μg of normal rabbit IgG (Sigma) antibody. Immunoprecipitated chromatin was reverse crosslinked and washed before DNA extraction. Polymerase A was used as a positive control while IgG was used as the negative control. Finally, the immunoprecipitated DNA and the corresponding non-immunoprecipitated DNA (input) was subjected to PCR using different set of forward and reverse primers, specific for the different putative binding regions, and analyzed on 1.5% agarose gel. The primers used for the different putative binding sites are listed in Supplementary Table 1.

### *In silico* Analysis of Promoter Binding Sites

UCSC MatInspector software was used to predict the putative promoter binding regions for Msx2. Primers were designed from these predicted regions for chIP followed by PCR amplification using these primers, using Primer 3 database.

### Imaging and Densitometric Quantification

The imaging for ISH was done using Olympus microscope while densitometric quantification of semi-quantitative RT-PCR bands were done using ImageJ (NIH, version 5).

### Statistics

Each cell culture experiment was replicated three times. For ISH, a minimum of 3–4 mice pups were used. Statistics was done using one-way ANOVA or students *t*-tailed test, wherever applicable using GraphPad prism (version 7, CA). *P* value of <0.05 was considered statistically significant.

## Results

### *Msx2* Differentially Regulates the Expression of Genes Involved in Amelogenesis

**FIGURE 1:**
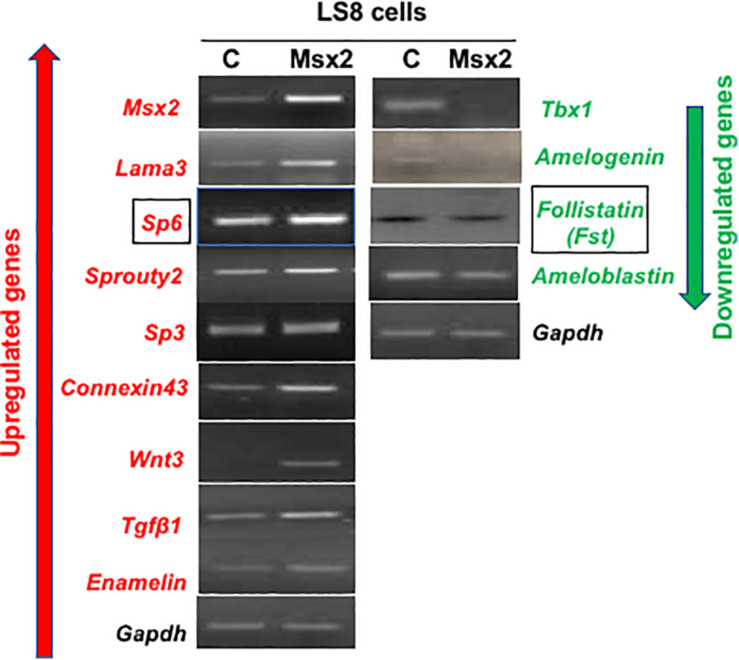
Screening of representative genes involved in amelogenesis: LS8 cells were transfected with pCMVtag2-Flag-*Msx2* then cultured for 48 h. Total RNA was isolated from the cells and subjected to reverse transcription PCR analysis. Upregulated genes are shown in red while downregulated ones are shown in green. The expression of *Sp6*, *Sp3*, *Sprouty 2*, *Connexin 43*, *Wnt3*, *Tgfb1* and *Enam* (*enamelin*), *Laminin 5 alpha 3* (*lama3*), as well as *Msx2* itself, is up-regulated in the *Msx2* overexpressing cells. In contrast, the expression of *Tbx1*, *Amel* (*amelogenin*), *Fst* (*Follistatin*) is downregulated in the *Msx2* overexpressing cells, while *Ambn* (*ameloblastin*) expression is partially diminished, almost not affected. *Gapdh* is the housekeeping gene. C, cells transfected with control vector only; Msx2, cells are transfected with pCMVtag2-Flag-Msx2.

There are several genes known to be involved in amelogenesis (Bei, 2009b; Wright et al., 2015). To test whether *Msx2* regulates the expression of some of these genes, we performed semi-quantitative RT-PCR after overexpressing *Msx2* in LS8 (Figure 1) and G5 cells (data not shown). The ameloblast-like cell LS8 cell line, derived from murine EO epithelium, and the G5 cell line, derived from rat dental epithelial derived ameloblast-lineage clone, both, constitute ideal cell systems to test gene regulation for the following reasons. (Chen et al., 1992; Xu et al., 2006; Abe et al., 2007; Ruspita et al., 2008). The LS8 and G5 “cells express many of the genes specific for amelogenesis, such as *ameloblastin*, *amelogenin*, and *enamelin*, at sufficiently high levels, they have been used for many *in vitro* studies of amelogenesis, including gene promoter analysis and LS8 cells, in particular, produce an enamel extracellular matrix that is similar to authentic enamel after treatment with peptide amphiphiles” (Zhou et al., 2000; Huang et al., 2008). After overexpressing *Msx2* in both cells lines, we show that the expression of *Sp6*, *Sp3*, *Sprouty 2*, *Connexin 43*, *Wnt3*, *Tgfb1* and *Enam* (*enamelin*), *Laminin 5 alpha 3* (*lama3*), as well as *Msx2* itself, is up-regulated in the *Msx2* overexpressing cells. In contrast, the expression of *Tbx1*, *Amel* (*amelogenin*), *Fst* (*Follistatin*) is downregulated in the *Msx2* overexpressing cells, while *Ambn* (*ameloblastin*) expression is partially diminished, almost not affected (Figure 1). These results further confirm previous results where we and others showed that *Msx2* is required for the regulation of *Lama3*, *Amel, Enam* and *Ambn* gene expression (Zhou et al., 2000; Bei et al., 2004; Ruspita et al., 2008; Molla et al., 2010; Bei, unpublished). We also show for the first time that *Msx2* is required for the regulation of *Sp6*, *Sp3*, *Sprouty 2*, *Connexin 43*, *Wnt3*, *Tgfb1*, *Tbx1*, and *Fst* (*Follistatin*) gene expression. For the purposes of this study, we will focus on *Sp6* and *Fst* regulation, only.

### The Expression of *Sp6* and *Fst* Is Modulated Early in Response to *Msx2*

**FIGURE 2:**
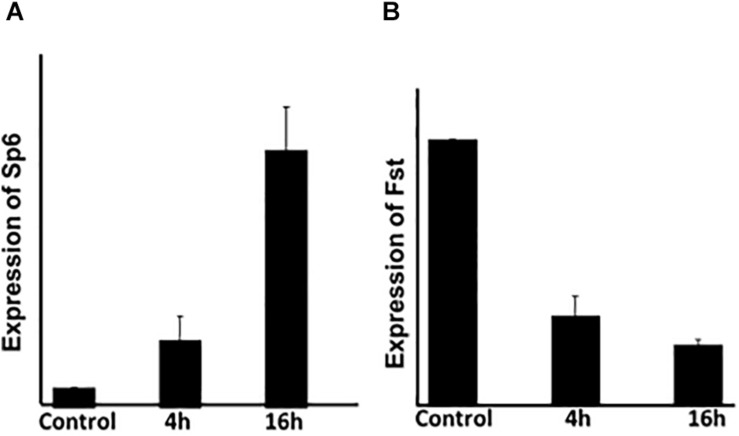
The expression of *Sp6* and *Fst* is modulated early in response to *Msx2:* LS8 cells were transfected with *Msx2* over-expression plasmid for two time points, 4 and 16 h. Total RNA was isolated from the cells and subjected to qPCR analysis. **(A)** *Sp6* and **(B)** *Fst* could be detected as early as 4h after transfection by real time qPCR. By 16 h, there was a significant increase in the expression of *Sp6* **(A)** with a corresponding decrease of *Fst* expression **(B)**. *Gapdh* is the normalizing gene. C, cells transfected with control vector only. The experiment was conducted three times in replicates of 3. **p ≤ 0.005.

The previous results indicate that *Sp6* expression is up-regulated while *Fst* expression is downregulated, upon *Msx2* overexpression. To determine the kinetics of *Sp6* and *Fst* (*Follistatin*) gene expression, we performed a time dependent assay to ascertain whether their expression is modulated early in response to *Msx2* upregulation (Figure 2). LS8 cells were transfected with *Msx2* over-expression plasmid for two time points, 4 and 16 hours. Total RNA was isolated from the cells and subjected to qPCR analysis. *Sp6* and *Fst* could be detected as early as 4h after transfection by real time qPCR (Figure 2). By 16 h, there was a significant increase in the expression of *Sp6* (Figure 2A) with a corresponding decrease of *Fst* expression (Figure 2B). We have not seen any significant response earlier than 4 h, thus these results indicate that both *Sp6* and *follistatin* are secondary early response genes to *Msx2* (response after 4 h of *Msx2* overexpression). These experiments have not been performed in the presence of the protein synthesis inhibitor, cycloheximide and thus, we do not know whether the secondary response of *Sp6* and *Fst* genes require *de novo* protein synthesis for transcription.

### Loss of Function of *Msx2* and *Sp6* in LS8 Ameloblast-Derived Cells

**FIGURE 3:**
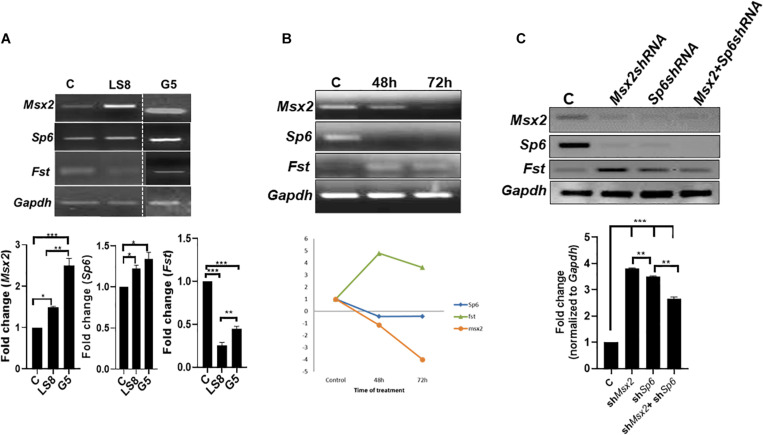
Loss of function of *Msx2* and *Sp6* in LS8 ameloblast-derived cells: **(A)** Both LS8 and G5 cells were overexpressed with *Msx2* over-expression plasmid. Representative RT-PCR showing *Sp6* is upregulated whereas *Fst* is downregulated in both cell lines after *Msx2* overexpression. Bottom panel shows densitometric quantification of the bands. **(B)** The knockdown of *Msx2* with siRNA relative to scrambled control shows downregulation of *Msx2* and *Sp6* and upregulation of *Fst*, in 48 and 72 h. RT-PCR results were normalized to *Gapdh* that served as an internal control and expression levels were relative to scrambled controls. **P < 0.01. Bottom panel shows a graph describing the trend of genes’ expression. **(C)** Lentiviral (ShRNA) gene knockdown assay, where *Msx2*-shRNA and *Sp6*-shRNA, and non-target-shRNA infected LS8 cells further confirm the siRNA results. qPCR results were normalized to *Gapdh.* Bottom panel shows densitometric quantification of the bands. Experiments were done in triplicates. **p ≤ 0.005; ***p ≤ 0.0001.

**FIGURE 4:**
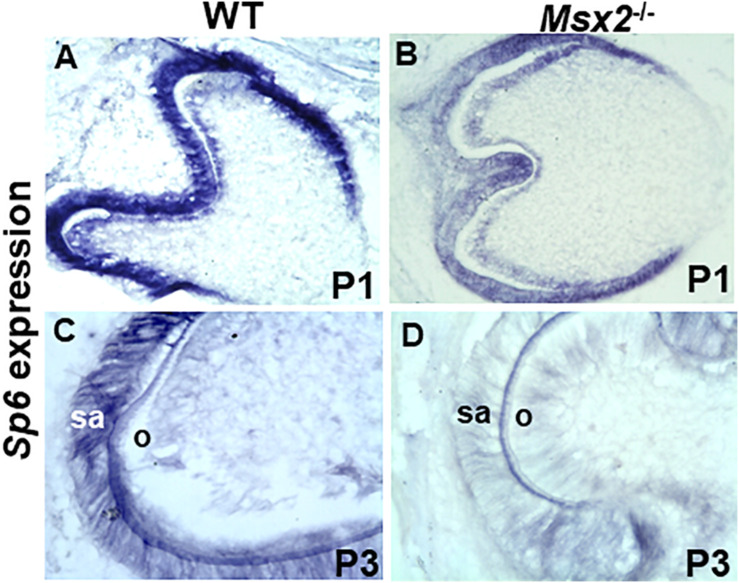
Msx2 is essential for *Sp6* gene expression during late stage tooth development: In situ hybridization analyses of transcripts in wild type **(A,C)** and *Msx2* deficient **(B,D)** first lower molar teeth at postnatal day P1 **(A,B)** and postnatal day P3 **(C,D)**. Expression of *Sp6* is reduced **(B)** relative to wild type **(A)**. In contrast, a dramatic reduction of *Sp6* expression, is observed in *Msx2* deficient ameloblasts **(D)** compared to wild type at P3 **(C)**. sa, secretory ameloblasts. Scale: X400 (*N* = 4).

We have shown that overexpression of *Msx2* in both LS8 (Figures 2, 3A) and G5 cells (Figure 3A) leads to a significant increase of *Msx2* in both cell lines-indicating that the transfection efficiency is quite successful-with concomitant increase of *Sp6* and decrease of *Fst* expressions (Figure 3A). To test whether the opposite holds true, we tested the effects of acute knockdown of Msx2 in LS8 ameloblast-derived cells, and compared to what happens in development where Msx2 is permanently absent in the *Msx2*-null mice (Figure 4). For the knock down experiment, we used siRNA technology in LS8 cells (Figure 3B). After 48 and 72 h transfection the cells were subjected to RT-PCR. We found that upon silencing of *Msx2*, *Sp6* was downregulated while *Fst* expression was upregulated, further suggesting that *Sp6* requires Msx2 for its expression and that Msx2 inhibits *Fst* expression (Figure 3B). In addition, we used lentiviral shRNA mediated approach to assess the direct effects of silencing *Msx2* and *Sp6* genes. Specifically, the LS8 cells were infected with mouse Msx2shRNA, or Sp6shRNA or both lentiviral transduction particles. qPCR shows that (i) Msx2shRNA or Sp6shRNA lentiviral transduction particles alone effectively reduce each other’s expression in LS8 cells, while they increase expression of *Fst* compared to control shRNA treated cells; (ii) that both Msx2shRNA plus Sp6shRNA lentiviral transduction particles abolished *Sp6* and *Msx2* expression in LS8 cells and increased *Fst* expression comparing with control shRNA treated cells (Figure 3C).

The loss of function along with the gain of function results show for the first time that Msx2 is required for *Sp6* expression and that Sp6 is required for *Msx2* expression. This result suggests that these two transcription factors may interact at the molecular level to control each other’s transcriptional output. We also show that Msx2 inhibits *Fst* expression and confirmed that Sp6 inhibits *Fst* expression (Ruspita et al., 2008). In addition, we show that Msx2 and Sp6 act synergistically to control the level of *Fst* inhibition, since when we silenced both Msx2 and Sp6, at the same time, the inhibition of *Fst* was less robust compared to silencing by *Msx2* or *Sp6* alone. The latter result is of particular interest as it suggests that the synergistic interaction of Msx2 with Sp6 may alleviate each other’s inhibitory effect on *Fst* by allowing one, or more than one, transcription activators to exert their function and thus regulating *Fst*’s gene dosage.

### Msx2 Is Essential for *Sp6* Gene Expression During Late Tooth Development

To determine whether *Msx2* is required for *Sp6* regulation and whether this requirement is associated with the defect in amelogenesis, *in situ* hybridization was performed in wild type and *Msx2* deficient mouse molar tooth germs at E.18.5, postnatal day 1 (P1) and postnatal day 3 (P3) (Figure 4). *Msx2* is expressed at very low levels by pre-ameloblasts (E18.5-P1) but is highly expressed in secretory ameloblasts (P3) (MacKenzie et al., 1992; Gritli-Linde et al., 2002; Bei et al., 2004; Bei, 2009a,b). Partial diminution of *Sp6* expression is observed in *Msx2* deficient tooth germs at P1 compared to wild type (Figures 4A,B). In contrast, a dramatic reduction of *Sp6* expression, is observed in *Msx2* deficient ameloblasts compared to wild type at P3, when ameloblasts are at their secretory stage (Figures 4C,D). This result indicates that *Sp6* requires *Msx2* for its expression in the secretory stage ameloblasts, and is consistent with the *in vitro* loss and gain of function studies.

*Follistatin (Fst)* starts to be co-expressed with *Msx2* at the early bell stage of tooth development. Although it seems to be expressed widely in the dental epithelial organ, its expression is more concentrated in the inner enamel epithelium (iee). As development proceeds however, and the inner enamel epithelium cells become pre-secretory ameloblasts and, later on, secretory ameloblasts, *Fst* ceases to be expressed (Wang et al., 2004; Bei, 2009b). In the absence of *Msx2*, *follistatin* is expressed throughout the dental epithelium at the bell stage and its expression is increased in the inner dental epithelium (data not shown). This result is consistent with the *in vitro* loss of function and gain of function studies.

### Msx2 Directly Binds to *Msx2* Recognition Sites on the *Sp6* Promoter

The loss and/or gain-of-function studies along with the *in vivo* experiments using the *Msx2* mouse mutants revealed that Msx2 is required for the expression of *Sp6* in the secretory ameloblasts, during amelogenesis. Computational sequence analysis of the nucleotides in the proximal 3.5 kb of the murine *Sp6* promoter region revealed the presence of 6 fully conserved Msx2 binding sites upstream from the transcription initiation site in the mouse (Supplementary Figure 1). To determine whether Msx2 binds to any of these sites and, therefore, directly regulates *Sp6*, chromatin immuno-precipitation was performed with exogenously expressed Msx2-FLAG. Immunoprecipitated chromatin fragments (IP samples) and non-immunoprecipitated samples (1% input) were subjected to PCR analysis using specific primers spanning the six binding sites. PCR amplifications showed that Msx2 binds directly to four out of six sites carrying the conserved motif (TAAT) in the endogenous promoter of the mouse *Sp6* gene (Supplementary Figure S1). This result demonstrates that Msx2 binds directly to the proximal *Sp6* promoter *in vitro*.

In sum, we show that (i) *Msx2* and *Sp6* exhibit overlapping expression in secretory ameloblasts; (ii) they regulate each other’s expression; (iii) *Msx2*, like *Sp6* alone or in coordination with *Sp6* inhibits *follistatin* expression; and (iv) *Msx2* binds directly to *Sp6* promoter, suggesting that *Sp6* is a direct target of *Msx2*.

Collectively, these results raise the possibility that the *Sp6* and *Msx2* transcription factors interact closely with each other and work in a concerted manner within a common molecular cascade to form part of a network of transcription factors that control ameloblast life cycle and amelogenesis.

## Discussion

Of the several transcription factors, *Msx2* and *Sp6* constitute key players of amelogenesis. Both, *Msx2* and *Sp6* mouse mutants, exhibit enamel hypoplasia, while humans with AI carry mutations in the human homologues of MSX2 or SP6 genes (Satokata et al., 2000; Bei et al., 2004; Suda et al., 2006; Nakamura et al., 2008; Utami et al., 2011; Babajko et al., 2014; Smith et al., 2020). These similarities in function indicate that these two transcription factors may reside in the same developmental pathway.

In this paper, we show that Msx2 and Sp6 transcription factors reside in the same developmental pathway and that they work in a coordinated manner to regulate the expression of *follistatin* (*Fst*), a signaling molecule that also controls enamel formation (Wang et al., 2004).

### Msx2 and Sp6 Transcription Factors Require Each Other to Exert Their Function

**FIGURE 5:**
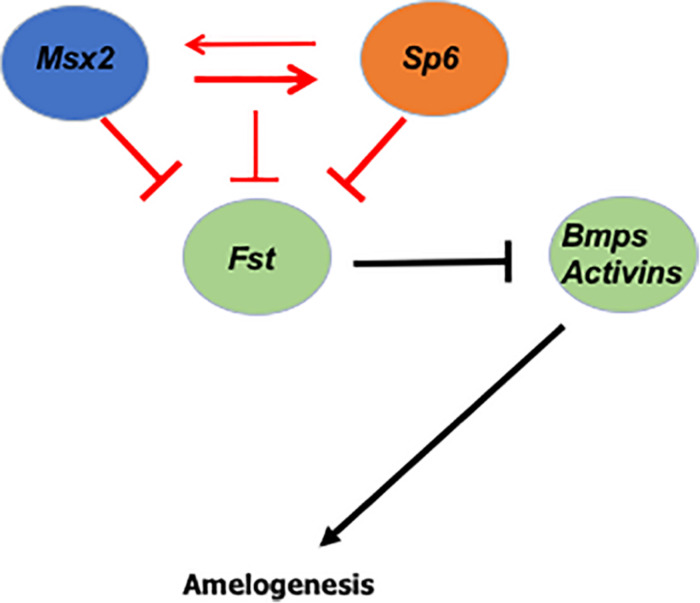
Predicted model of Msx2 and Sp6 function during amelogenesis: We show that the two transcription factors *Msx2* and *Sp6* regulate each other’s expression (red arrows) and that *Msx2* binds directly to *Sp6* promoter (thick red arrow), suggesting that *Sp6* is a direct target of *Msx2*. In addition, we show that *Msx2* alone, like *Sp6* (thick red inhibition lines) inhibits *follistatin* expression. In coordination with *Sp6* (thin red inhibition line) the inhibition of *follistatin* expression is less robust. Collectively, these results raise the possibility that the *Sp6* and *Msx2* transcription factors interact closely with each other and work in a concerted manner to form part of a network that controls ameloblast life cycle and amelogenesis, in a cell autonomous manner.

Our gain of function, loss of function, time dependent assay and *in vivo* data demonstrate that *Sp6* requires Msx2 for its expression (Figure 5). Our loss of function experiments indicate that *Msx2* also requires Sp6 for its expression, indicating that these two genes reside in the same genetic pathway and that these two transcription factors may interact at the molecular level to control each other’s transcriptional output (Figure 5). Characterization of *Sp6* promoter for Msx2 binding sites revealed six putative Msx2 binding sites and our ChIP experiments provided evidence that Msx2 binds directly to *Sp6* promoter to four out of six sites, suggesting that *Sp6* is a direct target for Msx2 and that Msx2 may promote *Sp6* expression directly acting as an activator of *Sp6* expression (Figure 5). Msx1 and Msx2 transcription factors are known to act as repressors (Catron et al., 1993, 1995; Zhang et al., 1997), but, consistent to our results recent findings in other developmental systems demonstrate that *Msx1* and *Msx2* may act as transcriptional activators, as well (Duval et al., 2014).

The fact that Msx2 binds directly to the *Sp6* promoter does not rule out the possibility that these two transcription factors may interact with each other physically and/or *in vivo* via a protein–protein interaction mechanism. Consistent with this idea, we have shown that Msx2 interacts *in vitro* and *in vivo* with another member of the Sp family of transcription factors, the Sp3 (Zhao et al., 2013). Interstingly, *Sp3* homozygous null mice, like *Sp6* null mice, exhibit a hypoplastic phenotype in both, dentine and enamel matrices (Bouwman et al., 2000).

Another interesting finding is that Msx2 seems to regulate its own expression. The gain of function experiments indicate that Msx2 is required for its own expression. This is consistent with other studies showing that Msx2 auto-regulates its promoter and simultaneously represses *Dlx2* transcription factor activation in LS8 ameloblast-like cells (Diamond et al., 2006). Here, we show that Msx2 is required for its own expression potentially via an autoregulation mechanism to activate the *Sp6* transcription factor. As transcription factors are known to operate sometimes through a feed-forward positive autoregulation mechanism, we can hypothesize that in this case Msx2 activates expression of *Sp6*, followed potentially by auto-regulatory binding to maintain expression of both genes at a certain level for specific time. To our knowledge, it is not known whether Sp6 operates through an auto-regulatory mechanism, like Msx2 does. Considering, however, that transfection of *Sp6* promoted dental epithelial cell differentiation into ameloblasts, by controlling the rate of proliferation of inner enamel epithelium (Nakamura et al., 2008; Ruspita et al., 2008), it would be interesting to see in the future, whether these two transcription factors sustain each other’s expression through positive autoregulation. Positive autoregulation is a process where a transcription factor either directly or indirectly activates its own expression, resulting in continuation of transcription in the absence of the factors that started its expression.

### *Follistatin* Expression Is Inhibited by, Both, Msx2 and Sp6 to Promote Amelogenesis

*In vivo* studies have clearly demonstrated that “*Follistatin* is essential for enamel-free area formation by preventing ameloblast differentiation” (Wang et al., 2004). “Overexpression of *follistatin*, a BMP inhibitor, in the epithelium abrogates ameloblast differentiation. The K14-*follistatin* mice lack enamel, the ameloblasts fail to form and they do not express enamel specific markers” (Wang et al., 2004). In contrast, in the *follistatin* knockout mice, functional ameloblasts differentiated on the normally enamel-free surface (Wang et al., 2004). In addition, “experiments on cultured tooth explants suggest that the mechanism by which *follistatin* prevents ameloblast differentiation is by inhibiting the ameloblast-inducing activity of BMP4 from the underlying odontoblasts. Thus, *follistatin* controls ameloblast differentiation in a cell-autonomous manner by integrating the effect of a non-cell-autonomous signal, that of BMP4 from odontoblasts” (Wang et al., 2004).

In the secretory ameloblasts, it is known that Sp6 inhibits *Fst* expression (Ruspita et al., 2008). Here, we show for the first time that, like Sp6, Msx2 inhibits *Fst* expression in the secretory ameloblasts and confirmed the previous result by Ruspita et al. (2008). In addition, we show that Msx2 and Sp6 act synergistically to control the level of *Fst* inhibition, since when we silenced both Msx2 and Sp6, at the same time, the inhibition of *Fst* was less robust compared to silencing by *Msx2* or *Sp6* alone (Figure 5). The latter result is of particular interest as it suggests that Msx2’s synergistic interaction with Sp6 may alleviate each other’s inhibitory effect on *Fst* by allowing a third or other transcription activators to exert their function and thus regulating *follistatin’s* final gene dosage (Figure 5).

### A Cell Autonomous Pathway of *Msx2*, *Sp6*, and *Fst* Operate to Ensure Enamel Formation

As mentioned above, the role of *follistatin* in preventing enamel to be formed by inhibiting the ameloblast-inducing activity of BMP4 from the underlying odontoblasts is well known (Wang et al., 2004). What this paper shows, is that Msx2 and Sp6 transcription factors coordinately function by regulating each other’s expression to ensure that the expression of *follistatin* is inhibited and that ameloblasts secrete enamel (Figure 5). *Follistatin* is expressed in the inner enamel epithelium to ensure proliferation of the cells and it does so by inhibiting the ameloblast-inducing activity of BMP4 from the underlying odontoblasts (Wang et al., 2004; Bei, 2009b). For enamel to be formed, *follistatin* needs to cease to be expressed, so that BMP4 from underlying odontoblasts is able to induce enamel formation. Thus, controlling the timing of *follistatin’s* switch, from on to off, is extremely important. In light of our recent findings, we propose that Msx2 and Sp6’s coordinated action controls either the cease of *follistatin’s* expression or the reduction of its level in order to promote enamel deposition, in a cell autonomous manner (Figure 5).

## Data Availability Statement

The raw data supporting the conclusions of this article will be made available by the authors, without undue reservation.

## Ethics Statement

The animal study was reviewed and approved by The Forsyth Institute and Massachusetts General Hospital Boston, Boston MA, United States.

## Author Contributions

IR and MB contributed to the concept and design of the study. IR, PD, YX, and MB contributed to the acquisition of data. IR, PD, SK, and MB prepared the figures. IR, PD, RD’S, and MB contributed to the data analysis and interpretation. IR, PD, and MB wrote the manuscript. IR, PD, MS, TN, RD’S, and MB contributed to the editing and the critical revision for intellectual content. All authors have approved the final version of the submitted manuscript.

## Conflict of Interest

The authors declare that the research was conducted in the absence of any commercial or financial relationships that could be construed as a potential conflict of interest.

## Supplementary Material

Supplementary Figure 1 Msx2 directly binds to *Msx2* recognition sites on the *Sp6* promoter: *In silico* model showing six potential *Msx2* binding sites (F1-R1: -1317 to -1323; F2-R2: -1731 to -1736; F3-R3: -1881 to 1886; F4-R4: -2109 to -2114, -2119 to -2124, -2141 to -2146; F5-R5: -2487 to -2493; F6-R6, position -2827 to -2833) on the *Sp6* promoter using MatInspector. The different binding sites are represented by green ellipses. Primers were designed from different promoter regions (blue arrows). After chromatin immunoprecipitation, samples from LS8 cells transfected with pCMV-Msx2-FLAG were PCR amplified; the binding region was directly amplified prior to immunoprecipitation (1% Input) and specifically amplified in the immunoprecipitated sample (anti-FLAG). (right panel). No amplification was detected in the normal mouse serum IgG-immuno precipitated sample (IgG; negative control). Pol A is the positive control. Band in the input lane shows endogenous binding while band in the sample lane shows binding after specific immunoprecipitation with FLAG tagged antibody after overexpression. The results show that *Msx2* binds only to four out of six predicted regions in the *Sp6* promoter.
