# Supplementary figures and images for: An Msx2-Sp6-Follistatin Pathway Operates During Late Stages of Tooth Development to Control Amelogenesis
Source: Front Physiol. 2020 Oct 26;11:582610. doi: 10.3389/fphys.2020.582610 (PMC7649293; doi:10.3389/fphys.2020.582610)

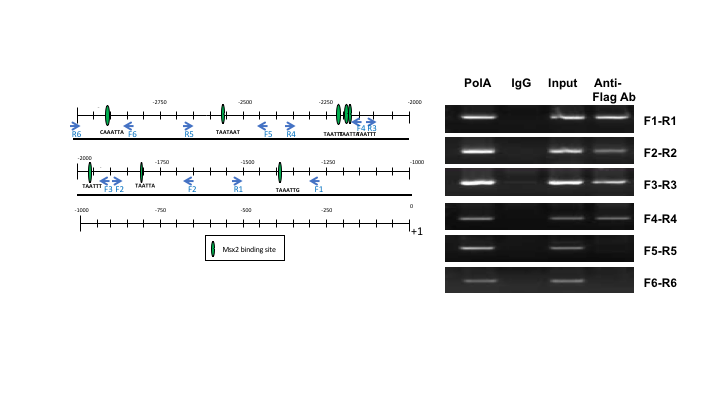

Supplement: Supplementary Figure 1 — Msx2 directly binds to Msx2 recognition sites on the Sp6 promoter: In silico model showing six potential Msx2 binding sites (F1-R1: -1317 to -1323; F2-R2: -1731 to -1736; F3-R3: -1881 to 1886; F4-R4: -2109 to -2114, -2119 to -2124, -2141 to -2146; F5-R5: -2487 to -2493; F6-R6, position -2827 to -2833) on the Sp6 promoter using MatInspector. The different binding sites are represented by green ellipses. Primers were designed from different promoter regions (blue arrows). After chromatin immunoprecipitation, samples from LS8 cells transfected with pCMV-Msx2-FLAG were PCR amplified; the binding region was directly amplified prior to immunoprecipitation (1% Input) and specifically amplified in the immunoprecipitated sample (anti-FLAG). (right panel). No amplification was detected in the normal mouse serum IgG-immuno precipitated sample (IgG; negative control). Pol A is the positive control. Band in the input lane shows endogenous binding while band in the sample lane shows binding after specific immunoprecipitation with FLAG tagged antibody after overexpression. The results show that Msx2 binds only to four out of six predicted regions in the Sp6 promoter. [file Image_1.TIFF]
