# Supplementary material for: An Msx2-Sp6-Follistatin Pathway Operates During Late Stages of Tooth Development to Control Amelogenesis
Source: Front Physiol. 2020 Oct 26;11:582610. doi: 10.3389/fphys.2020.582610 (PMC7649293; doi:10.3389/fphys.2020.582610)
Supplement: Supplementary file 2 [file Table_1.pdf]

Supplemental Table 1

Gene name	Forward primer	Reverse primer
Gapdh	**5'**CATTGACCTCAACTACATGG**3'**	**5'**CTCAGTGTAGCCCAGGATGC**3'**
Msx2	**5'**TCCGCCAGAAACAGTACCTC**3'**	**5'**GCAGCCATTTTCAGCTTTTC**3'**
Fst	**5'**TTTTCTGTCCAGGCAGCTCCAC**3'**	**5'**GCAAGATCCGGAGTGCTTCACT**3'**
Sp6	**5'**CCGGCAATGCTAACCGCTGTCTGTG**3'**	**5'**GGCTCAGTTGGAGGACGCCGAGCTG**3'**
Lama3	**5'**GGCCAAGTGGAGTTTGACAT**3'**	**5'**TGTCTCCTCCACAACTGCTG**3'**
Sprouty2	**5'**GGGTTAGGGGATTTGTGGTT**3'**	**5'**GCAATGTGGGTCTCCAACTT**3'**
Sp3	**5'**ACGCTCAGCAGGTTCAGATT**3'**	**5'**AGTTTGGCCAGGAAATGATG**3'**
Connexin45	**5'**GAACACGGCAAGGTGAAGAT**3'**	**5'**GAGCGAGAGACACCAAGGAC**3'**
Wnt3	**5'**GCGACTTCCTCAAGGACAAG**3'**	**5'**AAAGTTGGGGGAGTTCTCGT**3'**
Tgfβ1	**5'**TTGCTTCAGCTCCACAGAGA**3'**	**5'**TGGTTGTAGAGGGCAAGGAC**3'**
Enamelin	**5'**AACCAGTCCACAGGACCAAG**3'**	**5'**CCAGAGGCCCAATCTCATTA**3'**
Tbx1	**5'**ATGGGACGAGTTCAATCAG**3'**	**5'**GTACCGGTAGCCTTGTCAT**3'**
Amelogenin	**5'**CAAGAAATGGGGACCTGGATC**3'**	**5'**GCTGCCTTATCATGCTCTGG**3'**
Ameloblastin	**5'**CATGTCTTATGGAGCAAACCA**3'**	**5'**TCTTGCAGTGGAGAGCCTTCT**3'**
**Primers for Msx2 promoter binding Regions**		
**F1-R1**	**5'**TGCAGAAATTCTTCCCCTCT**3'**	**5'**AAAGCCAAACATCAGGGCTA**3'**
**F2-R2**	**5'**TATGTGTGCATTGGGCATTT**3'**	**5'**TTCATCTTGCAGGTCACAGC**3'**
**F3-R3**	**5'**TTGGCTCTTGCATGTGGATA**3'**	**5'**CAACATGGAGGAGGAACTCG**3'**
**F4-R4**	**5'**CTGAACTTCCTGCTCCACCT**3'**	**5'**CTTGTGGTTTCCACATGCAC**3'**
**F5-R5**	**5'**AGCTCCCTTGTCGAAGATCA**3'**	**5'**CACCATGCCTGACCTAAAGC**3'**
**F6-R6**	**5'**GCTTTTCC CACTTTCTCCT**3'**	**5'**CGACAAGGGAGCTAAAACCA**3'**
